# Influence of multiple freezing/thawing cycles on a structural, rheological, and textural profile of fermented and unfermented corn dough

**DOI:** 10.1002/fsn3.1193

**Published:** 2019-10-13

**Authors:** Sanabil Yaqoob, Huimin Liu, Chengbin Zhao, Meihong Liu, Dan Cai, Jingsheng Liu

**Affiliations:** ^1^ College of Food Science and Engineering Jilin Agricultural University Changchun China; ^2^ National Engineering laboratory for Wheat and Corn Deep Processing Changchun China

**Keywords:** fermented corn, freezing/thawing, rheology, structural, texture

## Abstract

In the current study, the impact of fermentation and freezing/thawing treatment on corn flour was studied. Fermentation revealed an increase (12%) in amylose content, while freezing reflected a loss of amylose. The results of scanning electron microscope (SEM) revealed more grooves, indentations, and the irregular shape of particles. Rapid Visco Analyzer (RVA) exhibited different pasting behavior on the dough. The molecular structure had similar profiles but showed several discernible absorbance at the different wavelengths. Differential scanning calorimetry (DSC) showed an increase in melting temperature range due to fermentation and freezing/thawing treatment attributed to more heterogeneous morphology. Overall, the results of this research showed the insight alterations that induce the changes in corn flour leading to improvement in some properties and it may enhance the acquaintance about the upright revolution in the profile of corn dough and its potential usage in industry and homes.

## 1 INTRODUCTION

Corn is considered to be the third most grown crop in the world, but most of the crop is transformed into fodder or ethyl alcohol and approximately only 5% is directly consumed by humans (Yang, Hui, Qiang, & Hua, 2017). The objective of modification is to enable the limited use of corn in the wide domain of applications. Currently, chemical, physical, and enzymatic modifications are widely used, but chemicals could be considered as hazardous in foodstuffs and it also worsens the taste as well. The chemical modifications mainly comprise silylation, esterification, carbamation, and hydrolysis. Physical modification such as extrusion and ultrafine technology is more reliable but expensive. In recent years, biotechnological modification of corn flour such as enzymolysis and fermentation is of keen interest due to high specificity and improved applicability. Yeast strains are tagged as generally recognized as safe (GRAS) by Food and Drug Administration (FDA) and are widely used in the food industry especially in baking and brewing products. As a matter of fact, amylases can be produced by only two genes, namely YIL099W and YIR019C, and both are glucoamylases (Kegg, 2013). Fermentation of cereals with yeast and lactic acid bacteria has been reported to purge the negative effects of the bran and improve texture, flavor, and structure of whole wheat and rye bread (Katina et al., 2005).

Shelf life is the most important concern in the food industry. Over the past few years, freezing technology was the most used method for fresh goods to increase shelf life. The effect of freezing on dough properties especially on its quality, rheology, and morphology was a field of active research. Freezing led to more moisture, recrystallization, synersis, and more ice growth (Ribotta, León, & Añón, 2003; Lee, Choi, Lee, & Min, 2003; Jacobson & BeMiller, 1998). Starch retrogradation with higher elastic values was observed in the sweet dough when frozen at −30 and −40°C (Meziani et al., 2011). Freezing water exerts pressure, results in compressed and crumbled granules due to phase transformation from water to ice crystals. Ultimately, a broader and coarser surface could be observed (Tao, Wang, Ali, et al., 2016; Tao, Wang, Wu, Wang, Wu, Jin, & Xu, 2016; Tao, Zhang, Zhang, Xu, Jin, & Xu, 2016). Frozen dough gradually deteriorated and led to altered morphology and lower texture retention ability. The altered structure results in leaching of lipid, proteins, and characterization of gelatinization and pasting behavior of dough (Vandeputte, Vermeylen, Geeroms, & Delcour, 2003). Corn flour was found to be supreme for frozen dough quality, where freezing brought about some stress on the granules and cause deterioration of granules and loss of integrity. Previous studies clearly indicated that freezing can cause coarse and broad granule which may be due to the formation of ice crystals that mainly depends on freezing rate. Slow freezing rate results in more ice growth, while rapid freezing showed less ice formation. Base on the literature, it is clear that the mechanism involved between interactions in frozen dough has not been profoundly studied. More research is thus needed to extensively understand the cause and effect relationships between frozen dough.

The objective of the present study was to characterize the changes in rheology, texture, morphology, structure, and thermal properties of fermented and unfermented corn dough with respect to multiple freezing/thawing cycles. Modification through fermentation has been used to improve the corn flour applicability induced by yeast at 35°C. The results obtained may provide novel insight about the corn granules and frozen dough deterioration, thereby providing a comprehensive data to improve the quality of a final thawed product.

## 2 MATERIALS AND METHODS

Corn grains (Jingke 1968) were provided by Jilin Agricultural University, China, for research purpose. Milling was done by laboratory miller (FW100; Taisite Co., Tianjin, China) and then passed through a mesh (80 number). Active dry yeast (Angel brand), sugar, and salt were purchased from local market. All other chemicals were of analytical grade.

### 2.1 Fermentation conditions

Yeast (0.5 g) was dispersed in mild water followed by continuous stirring. The ingredients of dough were 100 g corn flour, 60 ml water, 1 g salt, and 3 g sugar. All ingredients were thoroughly mixed to make a dough. The dough pieces were then placed in incubator at 35°C for 2 hr.

### 2.2 Preparation of dough for freezing/thawing cycles

Standard corn dough was prepared by adding water, the same fermented dough was also prepared, and all the samples were bagged in polythene packages. These bags were immersed in the freezer for storage at −25°C for 22 hr and then for two hours in a water bath at 25°C. This cycle was repeated for multiple times (0, 3, 5, and 7) and compared with the fresh dough. Sample codes are mentioned in Table 1.

**Table 1:** Sample codes

Sample code	Treatments
NF	Nonfermented flour
NF FTC 3	Nonfermented flour freezing/thawing cycle 3
NF FTC 5	Nonfermented flour freezing/thawing cycle 5
NF FTC 7	Nonfermented flour freezing/thawing cycle 7
FF	Fermented flour
FF FTC 3	Fermented flour freezing/thawing cycle 3
FF FTC 5	Fermented flour freezing/thawing cycle 5
FF FTC 7	Fermented flour freezing/thawing cycle 7

### 2.3 Pasting properties of flour

The pasting properties were analyzed by using Rapid Visco Analyzer (Perten) according to the method presented by Wani, Sogi, Wani, Gill, and Shivhare (2010). 3 g of flour was added in 25 ml of water and placed on RVA (Rapid Visco Analyzer) cup. The average values for peak viscosity (PV), trough viscosity (TV), final viscosity (FV), breakdown, setback, and pasting temperature (PT) were obtained for each sample.

### 2.4 Micromorphology of corn flour

Scanning electron micrographs (Phenom^TM^) of each sample were obtained at magnifications of ×5,000. Flour samples were fixed on the holders with double spread, and gold layer was sputtered on it and then scanned in a vacuum of 5 kv potential difference.

### 2.5 Textural analysis of corn dough

Fifty gram of corn flour was kneaded with 50 ml of water in order to make a standard dough by giving 60 min of rest at room temperature. The textural analysis comprises hardness, adhesiveness, springiness, cohesiveness, chewiness, gumminess, and resilience were measured by a textural analyzer (TA.XT plus). Maximum force applied can be considered as hardness. The conditions were as follows: test distance 10 mm, probe P/0.5, and test velocity 0.5 mm.

### 2.6 Chemical profile of dough

The amylose content was determined through the Megazyme kit as described by Williams, Kuzina, & Hlynka (1970and Williams, & Floyd (1970). Swelling power was measured by using Leach method, and 0.1 g sample was mixed with 10 ml water followed by heating at 60°C for 30 min with continuous stirring and then centrifugation at 460 g for 15 min (Leach, 1959). Water solubility was determined using Kainuma method, and 0.5 g sample was added in 10 ml water and heated for 30 min at 60°C followed by centrifugation for 10 min (Kainuma, 1967). The transmittance of samples was measured by the method of Lawal (2009), and 5% of sample suspension was heated at 100°C for 20 min with continuous stirring followed by cooling at room temperature. The samples were stored in a refrigerator, and transmittance was measured every 24 hr for 3 days at 650 nm against a water blank with a spectrophotometer. Synersis can be obtained as the amount of water released from the samples.

### 2.7 Rheological characterization of dough

Rheological properties were measured by using rheometer (Anton Paar, Modulus Compact Rheometer MCR‐302). Frequency sweep test was performed by adopting the following parameters, parallel plates (50 mm), a gap (1 mm), and temperature 25°C. The sample was placed on the plate, and excessive material was wiped off through the spatula. Silicone oil is added to the sample to avoid the evaporation, and 10‐min rest was given to equilibrate the stresses. First of all, the linear viscoelastic region was defined through a strain sweep test. Storage (G'), loss modulus (G"), and tangent delta (tan) were determined at constant shear strain and frequency range 0.1–10 Hz (Tao, Wang, Ali, et al., 2016; Tao, Wang, Wu, et al., 2016; Tao, Zhang, et al., 2016).

### 2.8 Molecular structure of corn flour

The change in the molecular structure of corn flours was recorded by scanning the flour pellet using FTIR (Bruker, Vertex‐70). 1 mg of sample was mixed with 100 mg KBr and then pressed to make tablets, and spectra were recorded from the range of 400–4,000 cm^‐1^ (Amir et al., 2013).

### 2.9 Thermal characterization of flour

Thermal properties of flour samples were analyzed by differential scanning calorimetry (DSC, TA Q 2000). Analysis conditions were as follows: 3 mg sample, 6 µl distilled water, and temperature 25–150°C at a constant rate of 10 min/°C. An empty aluminum pan was used as a reference. The respective parameters (the onset (T_O_), peak (T_P_), conclusion (T_C_), gelatinization temperature range (ΔT (T_C_–T_O_)), and gelatinization enthalpy (ΔH)) were determined with the use of universal analysis 2000 software (Reyes, Francisco, Angelica, Jaime, & Jose, 2016).

### 2.10 Statistical analysis

The data obtained for each parameter were analyzed in triplicate and subjected to statistical analysis by using Statistical Package Origin‐Pro 8.5. The experiment was performed under completely randomized design (CRD) and standard deviation, and the analysis of variance was applied to determine the level of significance followed by LSD.

## 3 RESULTS AND DISCUSSION

### 3.1 Pasting properties of flour

Pasting parameters of native, fermented, and freezing/thawing samples are summarized in Table 2. The whole modification did not change the overall shape of the Visco profile by RVA. Yeast fermentation results in significantly decreased pasting parameters such as PV, breakdown, PT, FV, and setback. This could be due to the macromolecule degradation such as starch, which significantly decreased during the process. The results obtained are closely consistent with the findings of Ilowefah, Bakar, Ghazali, Mediani, & Muhammad (2015). They explained the fact that acidification results in more fragility and breakage of starch granules. The results of pasting properties were also in agreement with previous researchers, who reported that setback and breakdown of rice flour decreased after lactic acid fermentation (Yang & Tao, 2008). The crystalline structure was formed due to amylopectin swelling that leads to more PV. Therefore, the alteration in amylopectin chain and polymerization are correlated with the decrease in PV and breakdown and leaching of polymers (Chiang & yeh, 2002).

**Table 2:** RVA parameters of various samples

Samples	Peak viscosity (RVU)	Trough viscosity (RVU)	Breakdown viscosity (RVU)	Final viscosity (RVU)	Setback viscosity (RVU)	Pasting temperature (^o^C)	Pasting time (min)
NF	1,783 ± 6	1,038 ± 7.50	745 ± 2.51	1,834 ± 7	796 ± 7.54	75.85 ± 0.11	4.4 ± 0.06
NF FTC 3	1,860 ± 7.23	1,110 ± 11	750 ± 1	2,136 ± 12.66	1,026 ± 8.50	74.2 ± 0.2	4.46 ± 0.07
NF FTC 5	1,890 ± 14.50	948 ± 4	740 ± 3.51	2,069 ± 10.01	856 ± 7.54	74.95 ± 0.15	4.50 ± 0.02
NF FTC 7	2,194 ± 5.50	1,089 ± 5.50	1,105 ± 5.50	2,147 ± 6.55	1,058 ± 3.05	74.3 ± 0.18	4.2 ± 0.04
FF	1,688 ± 15.01	948 ± 11.01	740 ± 2.08	1,804 ± 7.66	856 ± 7.02	75.1 ± 0.25	4.33 ± 0.04
FF FTC 3	1,715 ± 15.01	958 ± 12.2	757 ± 2.21	1,805 ± 5.13	847 ± 3.78	75.05 ± 0.07	4.26 ± 0.06
FF FTC 5	1,810 ± 3.51	948 ± 1.15	862 ± 5.68	1,798 ± 3.51	850 ± 1.52	75.15 ± 0.25	4.20 ± 0.01
FF FTC 7	2,004 ± 17	1,204 ± 15.01	800 ± 11.59	2,161 ± 10.26	957 ± 2.88	75 ± 0.11	4.33 ± 0.03

Abbreviations: NF, nonfermented flour; NF FTC 3, nonfermented flour freezing/thawing cycle 3; NF FTC 5, nonfermented flour freezing/thawing cycle 5; NF FTC 7, nonfermented flour freezing/thawing cycle 7; FF, fermented flour; FF FTC 3, fermented flour freezing/thawing cycle 3; FF FTC 5, fermented flour freezing/thawing cycle 5; FF FTC 7, fermented flour freezing/thawing cycle 7.

Freezing/thawing treatment caused a continuous increase (significantly) in PV, BV, FV, and SV as shown in Table 2. They were expected to be influenced by major constituents such as amylose, lipids, and protein contents. The role of amylopectin is starch swelling, whereas amylose maintains the swollen starch integrity and suppressed the swelling (Jane et al., 1999). Higher PV and BV mainly correlated with the lower amount of amylose content. That is why, freeze‐treated flour swelled greatly and degraded with more decrease in viscosity (Chung, Liu, Lee, & Wei, 2011). Leached amylose rapidly aggregated upon cooling. These junction zones of amylose were responsible for the formation of SV and FV (Barrera et al., 2013). Therefore, the extent of pasting properties of freeze/thawing processed flour was mainly (negatively/positively) correlated with the leaching of materials, damaged starch, and inner morphology. These aspects facilitated the interactions between molecules which involved in hydration.

### 3.2 Micromorphology of corn flour

**Figure 1:**
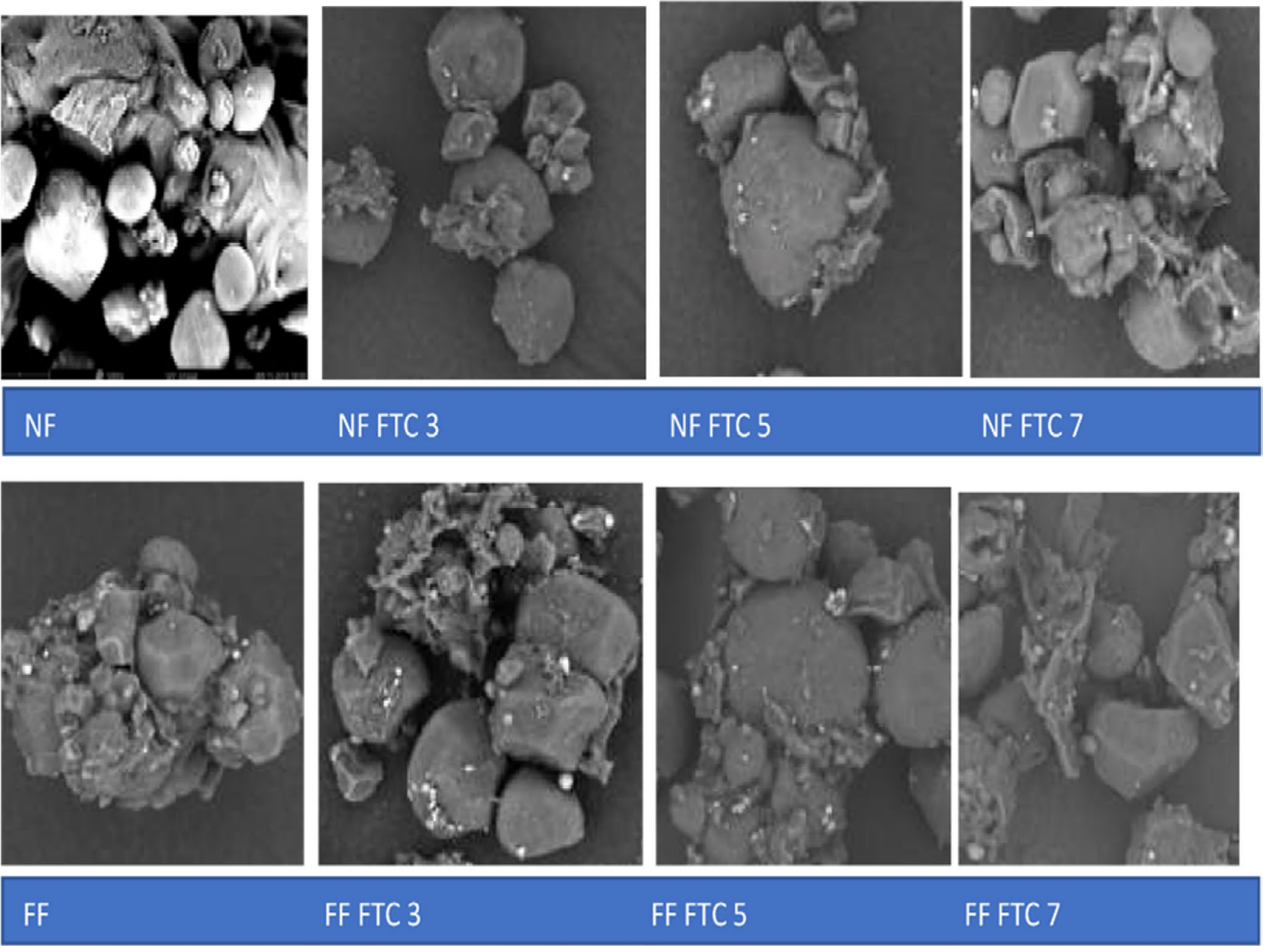
SEM micrographs of different samples. Each sample was obtained at magnifications of ×5,000 and scanned in a vacuum of 5 kv potential difference. NF, nonfermented flour; NF FTC 3, nonfermented flour freezing/thawing cycle 3; NF FTC 5, nonfermented flour freezing/thawing cycle 5; NF FTC 7, nonfermented flour freezing/thawing cycle 7; FF, fermented flour; FF FTC 3, fermented flour freezing/thawing cycle 3; FF FTC 5, fermented flour freezing/thawing cycle 5; FF FTC 7, fermented flour freezing/thawing cycle 7

SEM micrographs permit a direct observation of corn flour to explain the different properties of unfermented, fermented, and freeze/thawed samples. The micrographs of SEM are shown in Figure 1. Fermented corn flour had much smaller particles with sharp and irregular edges, while unfermented flour retained smooth surface (Di stasio, Vacca, Piciocchi, Meccariello, & Volpe, 2007). Fermented flour with smaller and irregular particles resulted in more water‐absorbing capacity, and less water retention capacity, which ultimately leads to more easy and compact dough and better rheology (Oh, Choi, Lee, Kim, & Moon, 2008). Freezing/thawing treatment directly imparts the action on the morphology of flour. The surface of freeze/thawed samples with multiple cycles displayed more grooves and shallow indentations. Multiple cycles expose more grooves and indentations. The reason could be the phase transformation, which resulted in suppressing and crumbling of granules all through freezing. The ice crystals occupied more space that resulted in disruption of granules. Szymonska, Krok, and Tomasik (2000) reported that deep freezing of starch gives rise to more folded and unorganized microstructure of granules. Similar observations were found in the high‐pressure chamber compressed starch (Vallons & Arendt, 2010).

### 3.3 Textural analysis of corn dough

There was a remarkable difference between unfermented and fermented dough for textural analysis of corn flour as shown in Table 3. The fermented dough has lower hardness, more cohesiveness, and chewiness as compared to the unfermented corn dough. Yang et al. (2017) studied the amylase‐rich fermented maize fortified flour and concluded better textured fermented flour which is in conformity with our study. Hardness is actually the maximum force applied on the dough, and lower hardness enables the easy processing of flour. However, multiple freezing/thawing can cause an increase in dough hardness, and these consequences are mainly related to starch retrogradation. The effect of freezing was more pronounced after multiple cycles which showed a significant increase in hardness of dough. Tao et al. (2015) studied the effect of freezing on bread and reported that after multiple freezing/thawing cycles volume was decreased.

**Table 3:** Textural properties of different samples

Treatment	Hardness (g)	Adhesiveness (g/sec)	Springiness (mm)	Cohesiveness	Gumminess (g)	Chewiness	Resilience
NF	87.98 ± 1.15^F^	−61.86 ± 0.66^C^	0.86 ± 0.04^E^	0.22 ± 0.01^G^	33.68 ± 1.13^F^	28.56 ± 0.56^H^	0.13 ± 0.04^F^
NF FTC 3	140.10 ± 1.34^C^	−68.67 ± 0.33^E^	0.88 ± 0.02^DE^	0.28 ± 0.04^F^	37.53 ± 0.34^E^	31.91 ± 0.31^G^	0.16 ± 0.01^E^
NF FTC 5	157.20 ± 6.41^B^	−83.77 ± 0.78^H^	0.90 ± 0.04^C^	0.32 ± 0.01^E^	41.44 ± 0.86^D^	34.57 ± 0.57^F^	0.19 ± 0.01^D^
NF FTC 7	168.07 ± 2.20^A^	−77.34 ± 0.48^G^	0.92 ± 0.02^C^	0.40 ± 0.02^D^	46.16 ± 0.28^C^	39.88 ± 0.36^E^	0.22 ± 0.04^C^
FF	68.01 ± 4.29^G^	−41.77 ± 0.31^A^	0.90 ± 0.01^CD^	0.46 ± 0.02^C^	47.08 ± 1.16^C^	43.78 ± 0.76^D^	0.18 ± 0.02^DE^
FF FTC 3	107.70 ± 7.44^E^	−64.90 ± 0.20^D^	0.86 ± 0.01^E^	0.50 ± 0.04^B^	53.33 ± 0.48^B^	46.30 ± 0.85^C^	0.25 ± 0.02^B^
FF FTC 5	125.17 ± 5.49^D^	−55.03 ± 0.37^B^	0.94 ± 0.04^B^	0.57 ± 0.04^A^	55.56 ± 0.41^A^	48.33 ± 0.26^B^	0.31 ± 0.04^A^
FF FTC 7	140.31 ± 4.48^C^	−72.90 ± 0.33^F^	0.97 ± 0.02^A^	0.58 ± 0.01^A^	55.75 ± 0.62^A^	52.11 ± 0.81^A^	0.31 ± 0.01^A^

Abbreviations: NF, nonfermented flour; NF FTC 3, nonfermented flour freezing/thawing cycle 3; NF FTC 5, nonfermented flour freezing/thawing cycle 5; NF FTC 7, nonfermented flour freezing/thawing cycle 7; FF, fermented flour; FF FTC 3, fermented flour freezing/thawing cycle 3; FF FTC 5, fermented flour freezing/thawing cycle 5; FF FTC 7, fermented flour freezing/thawing cycle 7.

Different superscript letter showed significant difference at *p* ≤ .05.

### 3.4 Chemical profile of dough

The chemical composition of fermented, unfermented, and freeze/thawed samples is shown in Tables 4 and 5. Fermented corn possesses the higher amount of amylose content corresponding to 54.39%, while unfermented corn contains 48.59% amylose content. The increase in amylose content may be due to the hydrolysis of amylopectin into amylose. Similar results were found with fermentation of cocoyam flour (Oke & Bolarinwa, 2011). Chemical profile of Chinese chestnut also showed the change in rheological properties due to different amylose content (Yu et al., 2016). However, freezing can cause a decrease in amylose content which may be due to the phase transformation and mechanical forces or pressure exerted on granules. In this manner, thawing resulted in leaching of the constituents due to broader granular pathway and results in the slight increase in crystallinity. Swelling power and water solubility represent the interaction between crystalline and amorphous regions (Takizawa, Silva, Konkel, & Demiate, 2004). Moreover, these properties are influenced by amylose and amylopectin and tend to have strong harmony among them (Chan, Bhat, & Karim, 2009). The lower swelling power and solubility attributed to the higher amylose content which leads to the adjustment of the internal structure of granules. Synersis is defined as the amount of water released after storage. Fermentation improved the synersis of corn dough due to the change in gel structure. Starch retrogradation is indirectly involved in the breakdown of granules with the amorphous and crystalline regions, which were able to increase its water holding capacity, hence ultimately improved the synersis (Mirmoghtadaie, Kadivar, & Shahedi, 2009). The transmittance of corn dough increased with the increase in storage time. At the start day, fermented flour showed lower transmittance than unfermented flour which increases with the passage of time. Unfermented corn dough has an opaque paste with low light transmittance, while fermented dough has a higher transmittance. This may be due to the retrogradation property of cornstarch. Freezing/thawing attributed to the leaching of constituents and higher transmittance, which is in agreement with the results of synersis as reported by Amir et al. (2013).

**Table 4:** Chemical profile of samples

Treatment	Amylose content (%)	Swelling power (g/g)	Water solubility (g/g)	Synersis (%)	Absorbance 1,045/1,022 ratio
NF	48.59 ± 0.04^E^	6.51 ± 0.09^D^	6.12 ± 0.03^D^	15.56 ± 0.01^A^	0.70 ± 0.01^E^
NF FTC 3	46.73 ± 0.12^F^	6.80 ± 0.06^C^	6.54 ± 0.05^C^	9.88 ± 0.04^C^	0.81 ± 0.04^CD^
NF FTC 5	43.26 ± 0.10^G^	7.13 ± 0.07^B^	6.97 ± 0.03^B^	7.82 ± 0.01^D^	0.89 ± 0.01^B^
NF FTC 7	43.29 ± 0.06^G^	7.47 ± 0.0.06^A^	6.21 ± 0.06^A^	7.03 ± 0.02^E^	0.97 ± 0.02^A^
FF	54.39 ± 0.10^A^	5.77 ± 0.10^F^	4.96 ± 0.05^G^	10.10 ± 0.02^B^	0.78 ± 0.02^D^
FF FTC 3	51.91 ± 0.05^B^	5.56 ± 0.09^G^	5.24 ± 0.07^F^	5.88 ± 0.04^F^	0.88 ± 0.04^BC^
FF FTC 5	50.16 ± 0.04^C^	6.32 ± 0.11^E^	5.97 ± 0.06^E^	4.36 ± 0.04^G^	0.99 ± 0.04^A^
FF FTC 7	48.81 ± 0.05^D^	6.67 ± 0.07^CD^	6.21 ± 0.08^D^	4.24 ± 0.01^G^	0.99 ± 0.01^A^

Abbreviations: NF, nonfermented flour; NF FTC 3, nonfermented flour freezing/thawing cycle 3; NF FTC 5, nonfermented flour freezing/thawing cycle 5; NF FTC 7, nonfermented flour freezing/thawing cycle 7; FF, fermented flour; FF FTC 3, fermented flour freezing/thawing cycle 3; FF FTC 5, fermented flour freezing/thawing cycle 5; FF FTC 7, fermented flour freezing/thawing cycle 7.

Different superscript letter showed significant difference at *p* ≤ .05.

**Table 5:** The transmittance of corn dough

Transmittance				
Treatment	0 day	1 day	2 day	3 day
NF	1.47 ± 0.01	1.81 ± 0.02	1.89 ± 0.03	1.97 ± 0.03
NF FTC 3	1.54 ± 0.01	1.84 ± 0.03	1.84 ± 0.01	1.87 ± 0.03
NF FTC 5	1.62 ± 0.02	1.63 ± 0.04	1.84 ± 0.01	1.76 ± 0.03
NF FTC 7	1.65 ± 0.04	1.74 ± 0.02	1.67 ± 0.04	1.83 ± 0.03
FF	1.39 ± 0.02	1.50 ± 0.03	1.51 ± 0.03	1.58 ± 0.06
FF FTC 3	1.67 ± 0.02	1.73 ± 0.04	1.75 ± 0.03	1.77 ± 0.03
FF FTC 5	1.89 ± 0.04	1.70 ± 0.07	2.11 ± 0.31	2.39 ± 0.06
FF FTC 7	1.95 ± 0.04	2.04 ± 0.06	2.19 ± 0.05	2.44 ± 0.05

Abbreviations: NF: nonfermented flour, NF FTC 3: nonfermented flour freezing/thawing cycle 3, NF FTC 5: nonfermented flour freezing/thawing cycle 5, NF FTC 7: nonfermented flour freezing/thawing cycle 7, FF: fermented flour, FF FTC 3: fermented flour freezing/thawing cycle 3, FF FTC 5: fermented flour freezing/thawing cycle 5, FF FTC 7: fermented flour freezing/thawing cycle 7.

### 3.5 Rheological characterization of dough

**Figure 2:**
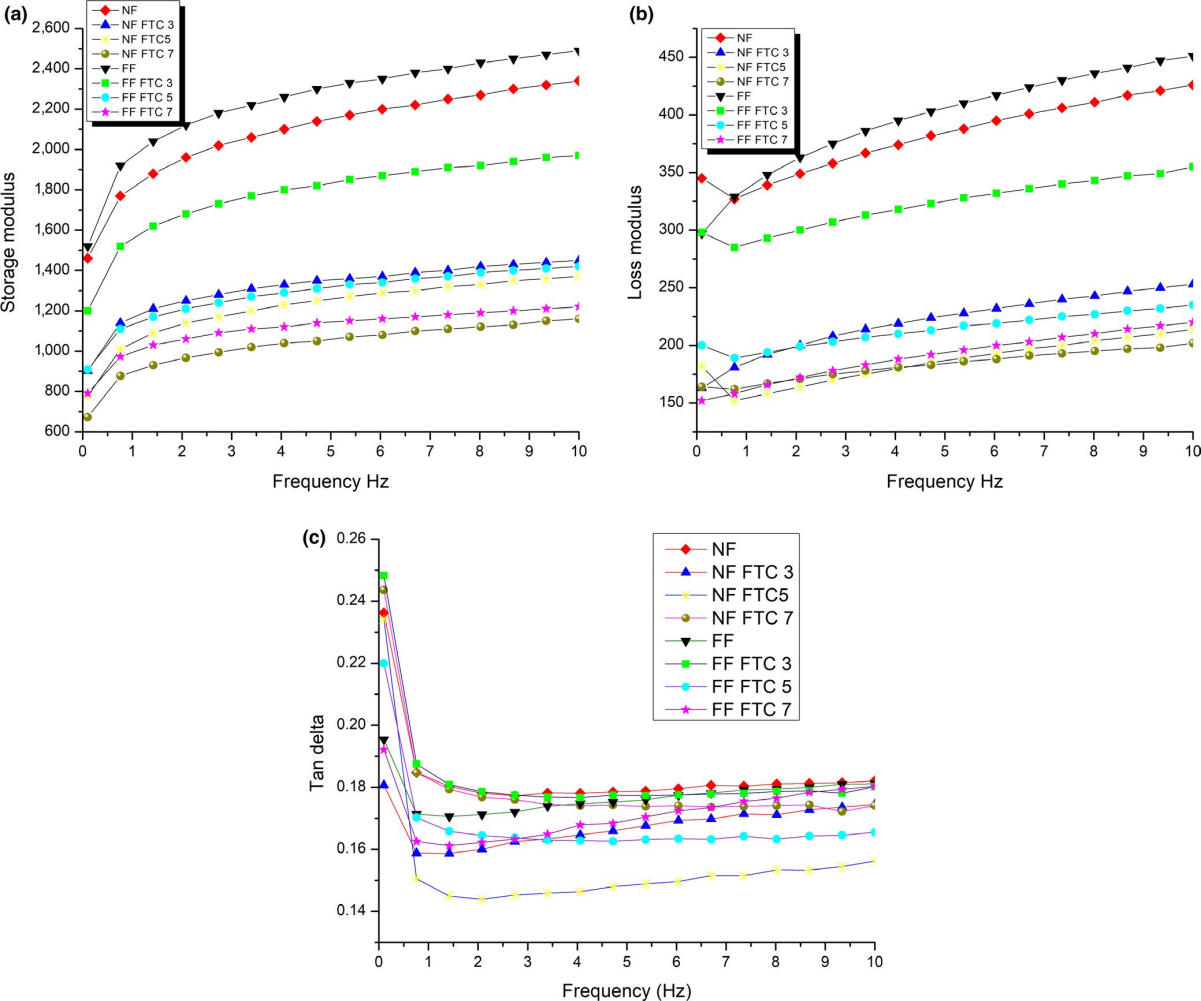
Rheological characterization of dough. (a) Storage modulus, (b) loss modulus, and (c) tan delta of dough. Parameters of frequency sweep test were as follows: parallel plates (50 mm), a gap (1 mm), and temperature 25°C. Storage (G'), loss modulus (G"), and tangent delta (tan) were determined at constant shear strain and frequency range 0.1–10 Hz

Rheology was the most important factor governing the proper manufacturing of dough. Quality and processability of dough ingredients have been studied by a frequency sweep test. Broader comprehension of dough rheology was essential to control and produce the high‐quality product (Letang, Piau, & Verdier, 1999). The storage modulus, loss modulus, and tan delta comprise the parameters for rheology of dough as shown in Figure 2. Both storage modulus and loss modulus are directly proportional to frequency. Loss modulus is always inferior to storage modulus, indicating that dough was more elastic than viscous (Narsimhan, 1994). The fermented dough exhibited higher storage and loss modulus than unfermented dough. The highly viscous material has been formed during gelatinization due to the leaching of amylose and amylopectin chains from the granules and severely affected the starch configuration through enzymatic hydrolysis (Debet & Gidley, 2007). While freezing/thawing treatment results in a slight decrease in storage and loss modulus while almost same tan delta. Previous research has reported that water expanded due to phase transformation and a coarse surface is formed that could accelerate the absorption of water which plays a pivot role in determining the rheology of dough (Tao, Wang, Ali, et al., 2016; Tao, Wang, Wu, et al., 2016; Tao, Zhang, et al., 2016). Enzymatic hydrolysis more specifically the amylolytic enzymes led to the partial fragmentation of flour, which in turn affects the dough rheology.

### 3.6 Molecular structure of corn flour

**Figure 3:**
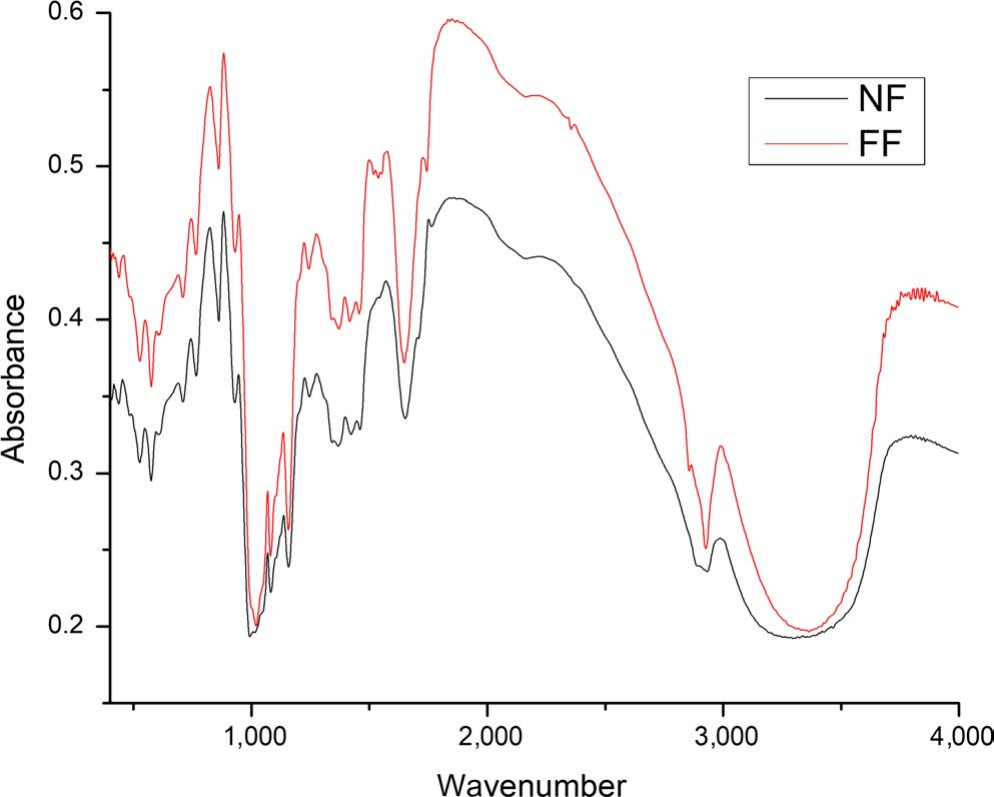
FTIR spectra of fermented and nonfermented flour. 1 mg of sample was mixed with 100 mg KBr and then pressed to make tablets, and spectra were recorded from the range of 400–4,000 cm^‐1^. NF, nonfermented flour; FF, fermented flour

The FTIR spectra of fermented and nonfermented flour had almost similar profiles (Figure 3). The fingerprint region displayed several discernible absorbances mainly attributed to C‐O bond stretching. There were some peaks arose at the start mainly at 568 and 765 cm^‐1^ as a result of fermentation indicating the vibration of CH_2_. The major constituent of corn flour is starch and it is a polymer of glucose, and every glucose unit contains CH_2_. As discussed previously, that fermentation can cause hydrolyzation results in more amylose content, lower cross‐linking, and better rheological properties (Peressini, Bravin, & Sensidoni, 2004). Moreover, the crystalline and amorphous structure is linked to 1,045 and 1,022 FTIR absorbance bands. The absorbance ratio has been proposed to magnify conformational changes in these regions (van Soest, Tournois, Wit, & Vliegenthart, 1995). The intensity ratio, as an indication of structural conformational changes, was significantly increased in fermented flour and also due to the freezing/thawing treatment as shown in Table 3. Fermentation increases the crystallinity, and decreases the amorphous material by altering the structure. Meziani et al. (2011) studied the conformational changes in structure due to freezing/thawing and concluded that it alters the structure and induces reorganization of double helices and its extent of interaction.

### 3.7 Thermal characterization of flour

The gelatinization properties of flour samples determined by DSC are summarized in Table 6. The onset (T_O_), peak (T_P_), conclusion (T_C_), gelatinization temperature range (ΔT (T_C_–T_O_)), and gelatinization enthalpy (ΔH) of nonfermented were 68.61°C, 73.74°C, 78.78°C, 10.17°C, and 2.72 J/g, while those of fermented flour were 65.59°C, 75.80°C, 86.48°C, 20.89°C, and 4.97 J/g, respectively. Results showed an increase in gelatinization temperature range due to fermentation and freezing/thawing treatment attributed to more heterogenous morphology of fermented flour than nonfermented. Fermentation reflects an increase in gelatinization enthalpy corresponding to more ordered granules, while freezing/thawing cycles lowers the gelatinization enthalpy (Utrilla‐Coello et al., 2014). The more freezing/thawing cycles attributed to higher onset (T_O_), peak (T_P_), and conclusion (T_C_) temperature (Tao, Wang, Ali, et al., 2016; Tao, Wang, Wu, et al., 2016; Tao, Zhang, et al., 2016). Water absorption is another factor effecting the thermal properties in freezing/thawing treatment because it provides a limited amount of water in the dough, thus directly effect the thermal property.

**Table 6:** Thermodynamic parameters of various samples

Treatment	T_O_ (°C)	T_P_ (°C)	T_C_ (°C)	ΔT (T_C_–T_O_) (°C)	ΔH (J/g)
NF	68.61 ± 0.07^E^	73.74 ± 0.13^E^	78.78 ± 0.14^D^	10.17 ± 0.07^G^	2.72 ± 0.15^E^
NF FTC 3	67.86 ± 0.13^F^	73.53 ± 0.17^E^	80.17 ± 0.14^C^	12.30 ± 0.03^F^	1.80 ± 0.07^F^
NF FTC 5	70.29 ± 0.17^C^	75.59 ± 0.25^C^	86.47 ± 0.14^B^	16.18 ± 0.02^DE^	1.41 ± 0.11^G^
NF FTC 7	71.63 ± 0.21^A^	78.24 ± 0.10^A^	87.74 ± 0.18^A^	16.11 ± 0.39^E^	1.00 ± 0.07^H^
FF	65.59 ± 0.09^G^	75.80 ± 0.15^C^	86.48 ± 0.16^B^	20.89 ± 0.06^A^	4.97 ± 0.15^A^
FF FTC 3	68.34 ± 0.16^E^	75.21 ± 0.17^D^	86.64 ± 0.14^B^	18.29 ± 0.22^B^	4.52 ± 0.09^B^
FF FTC 5	69.72 ± 0.19^D^	77.87 ± 0.15^B^	86.50 ± 0.16^B^	16.78 ± 0.02^C^	4.02 ± 0.05^C^
FF FTC 7	70.94 ± 0.13^B^	78.12 ± 0.09^AB^	87.46 ± 0.09^A^	16.52 ± 0.22^CD^	2.98 ± 0.09^D^

Abbreviations: NF, nonfermented flour; NF FTC 3, nonfermented flour freezing/thawing cycle 3; NF FTC 5, nonfermented flour freezing/thawing cycle 5; NF FTC 7, nonfermented flour freezing/thawing cycle 7; FF, fermented flour; FF FTC 3, fermented flour freezing/thawing cycle 3; FF FTC 5, fermented flour freezing/thawing cycle 5; FF FTC 7, fermented flour freezing/thawing cycle 7.

Different superscript letter showed significant difference at *p* ≤ .05.

## 4 CONCLUSIONS

The study concluded that the fermentation and multiple freezing/thawing treatment modified the dough quality and has better applicability. These modifications exerted a positive impact on physicochemical, structural, rheological, morphological, and thermal properties of corn dough. The morphological features showed irregular shape, more grooves, and shallow indentations lead to a more compact dough with better rheology. The amylose content was higher in fermented flour; however, these significantly decreased due to freezing/thawing. Freezing interferes with the overall profile of dough due to pressure exerted by ice crystals due to phase transformation and leaching of constituents such as protein, starch, and lipids. The results provided sufficient information and deeper insight to understand the improvement in corn flour applicability due to fermentation and freezing/thawing treatment, and it may enhance the acquaintance about the upright revolution in the profile of corn dough and its potential usage in industry and homes.

## CONFLICT OF INTEREST

The authors declare no conflict of interest.

## ETHICAL APPROVAL

This study does not involve any human or animal testing, and written informed consent was obtained from all study participants.
